# Optimization of X-axis servo drive performance using PSO fuzzy control technique for double-axis dicing saw

**DOI:** 10.1038/s41598-023-47663-y

**Published:** 2023-11-25

**Authors:** Weifeng Cao, Peiyi Zhang, Qingtao Mi, Yahui Sun, Jun Shi, Wanyong Liang

**Affiliations:** 1 College of Electrical and Information Engineering, Zhengzhou University of Light Industry, Henan, 450000 China; 2 Zhengzhou Guangli Ruihong Electronic Technology Co., Zhengzhou, 450000 China

**Keywords:** Engineering, Nanoscale devices

## Abstract

The dicing saw is a critical piece of equipment in IC processing, primarily used to cut wafers. Due to the high spindle speed, even small errors in the cutting process can result in wafer chipping or cracking. Therefore, the dicing saw requires a high degree of accuracy and stability. In this paper, the accuracy of the X-axis servo response was simulated using an Israeli ADT-8230 dual-axis abrasive wheel dicing saw. The study introduces a novel approach by using a fuzzy controller instead of the traditional position loop proportional integral (PI) controller. In addition, a two-input, two-output fuzzy rule is used for on-line correction of the position loop PI parameters. A heuristic algorithm is used to optimise the position loop fuzzy controller parameters. The quantization and proportionality factors are rectified using Particle Swarm Optimisation (PSO) algorithm and Genetic Algorithm (GA) respectively. By comparing the performance of the PSO fuzzy and GA fuzzy controllers, the optimal control method is derived. The proposed method is validated by simulation in the MATLAB/Simulink development environment using real ADT-8230 servo data. Experimental results show that the PSO-fuzzy structured controller reduces the position control error by 11.8%, improves the tracking performance by 26% and reduces the torque pulsation by 23%. Therefore, in future research, more advanced search algorithms should be further combined to improve the servo accuracy of the dicing saw.

## Introduction

**Figure 1:**
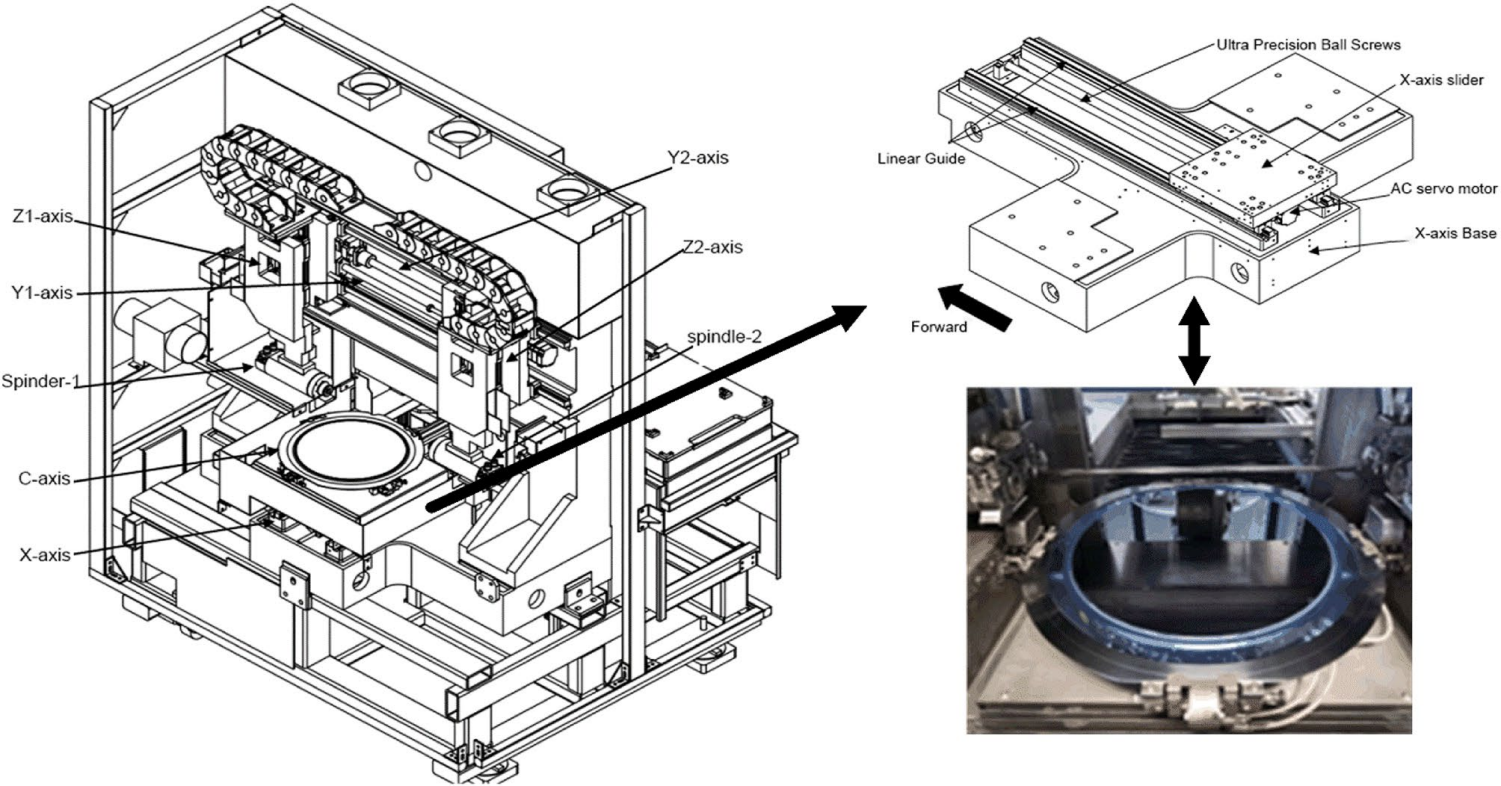
The mechanical composition of each axis of the ADT-8230 dicing saw and X-axis structure.

Dicing saw is a process in the semiconductor back-end packaging, which is widely used in the field of optical, precision machinery, microelectronic devices^1,2^. The ADT-8230 is mainly used for cutting various semiconductor substrate materials^3^. As the spindle speed of the wafer dicing saw is very high, slight deviation will cause the wafer to chip and crack, so the cutting quality of the dicing saw will directly affect the chip quality. Therefore, the servo output control should be very accurate. Figure 1 shows the mechanical composition of each axis of the ADT-8230. The servo axes in the ADT-8230 dicing system are mainly X-axis, Y-axis and C-axis, which also include stepping system Z-axis and high-speed aerostatic electrical spindle. The role of the X-axis is to move the wafer back and forth to complete the cutting. Due to the advancement of manufacturing technology, physical breakthroughs and improved integration, the chip size is being reduced, and with the continuous reduction of chip size, the dicing saw needs to have higher cutting efficiency and more accurate cutting precision to meet the manufacturing needs of small-sized chips.

The X-axis servo system consists of various mechanical and electrical components. The mechanical components include linear guides, ultra-precision ball screws, sliders and bases. These components provide stability and precision in the movement of the servo system. On the other hand, the electrical components of the X-axis servo system consist of a Yaskawa servo driver and a rotary AC servo motor. The servo motor is a three-phase AC permanent magnet synchronous motor (PMSM). This type of motor is widely used in high-precision industrial control applications due to its advantageous characteristics^3,4^, including relatively simple structure, light weight, small size, low loss and high efficiency^5,6^. However, it is important to note that the PMSM is a complex object with multiple variables, strong coupling, non-linearity and variable parameters. These characteristics can pose challenges in maintaining system performance, especially when the system is subjected to external disturbances^7^.

At present, the position control of the X-axis of the dicing saw mainly uses the proportional integral (PI) controller. The traditional PI control method, although the parameter adjustment is convenient, there is a certain degree of control accuracy, for the establishment of an accurate mathematical model of the system is very suitable. But in actual industrial production is difficult to establish an accurate mathematical model, most of the non-linear and uncertainty. Conventional PI control due to model inaccuracy and other reasons, most of the cases are tuned by empirical method, which is time-consuming, and difficult to find the optimal parameters. In order to achieve better control performance and meet the requirement of cutting accuracy, the introduction of intelligent control theory and improved excitation controller are recognised as both reliable and economically efficient methods. In servo control systems, there are various control algorithms such as artificial neural networks, robust, iterative and adaptive control algorithms^8,14^. These control algorithms are widely used in the field of high-precision control. However, these control methods usually require accurate device models to ensure control performance, and most of them improve the robustness of the control system at the expense of control accuracy. Therefore, the above methods are not well suited to the needs of the X-axis servo system. Therefore, in order to achieve high-performance control of the X-axis of the scribing machine, it is necessary to explore a simple control method with a simple structure, which can ensure the control accuracy and has a good anti-jamming ability of the system.

In recent years, fuzzy logic controllers (FLC) have been widely used in the field of complex nonlinear industrial control. Unlike traditional PI control, fuzzy control does not depend on the precise mathematical model of the controlled object, and it is an easy to understand, less susceptible and more desirable nonlinear controller. Therefore, fuzzy control has been widely used in a variety of scenarios, especially in the field of permanent magnet synchronous motor control. Kim et al.^15^ proposed a fuzzy PID control algorithm to solve the problem of PI control with large overshoot and long setting time when switching speed. Chao et al.^16^ developed the fuzzy PID controller with fewer parameters by combining traditional PID and optimal fuzzy PID controller design. Tavoosi et al.^17^ analyzed the fuzzy system rules and PID parameters so that the parameters can be adjusted online to minimize the fitness function. Dhandayuthapani^18^ demonstrated that the fuzzy controller system has better response than PI control. Wang^19^ studied the design of fuzzy adaptive PID control system is to avoid lengthy fuzzy system. Wang et al.^20^ combined the advantages of fuzzy PID and predictive function control to solve the undesirable performance of traditional PID control. However, there are drawbacks to fuzzy control; one of the main drawbacks of fuzzy controllers is that there are too many parameters to adjust. Especially when the parameters are set, it is very difficult for it to be given because there is no relevant reference.

In order to improve the transient and steady state behaviour of fuzzy controllers, several strategies and methods for parameter tuning have been proposed. One notable approach is the use of heuristics and meta-heuristics, which have greatly improved and simplified the optimisation of complex problems that were previously difficult or even impossible to solve. These heuristic and meta-heuristic algorithms are typically designed to simulate natural evolution by modelling the underlying principles of evolution found in nature. They use the concept of natural evolution to develop algorithms that efficiently search for optimal or near-optimal solutions within large solution spaces. These algorithms include simulated annealing algorithms, fuzzy control algorithms, genetic algorithms and neural network learning algorithms^21,24^, etc. Although these methods have improved the control effect to a certain extent, they also have shortcomings: genetic algorithms are slow to evolve and easy to mature prematurely; neural networks are prone to fall into the local optimum; simulated annealing algorithms are long in execution time, slow in convergence, and algorithms are affected by the initial value of the algorithm, there is parameter sensitivity and other problems. PSO and GA have the advantages of easy implementation, high accuracy, fast convergence, etc., can be a good match for PID excitation controller^25^. Zaway I et al, tuned fractional order proportional-integral-derivative (FOPID) controller using GA approach with two objective functions to minimise the error, energy and start-up torque to improve the control performance and robustness^26^. Razali et al, used GA to optimise the gains of fuzzy PID controller and the results proved that the fuzzy PID optimised by GA gives more accurate values^27^.Tang^28^ presented to optimize the control parameters of the controller using the PSO to obtain the optimal response error. Feng^29^ proposed optimizing the PID controller coefficients using an improved PSO algorithm to obtain the optimal trajectory tracking accuracy. Chang^30^ presented an improved PSO algorithm for find the optimal PID controller gain. Reath et al.^31^ analyzed the use of GA algorithm to improve the output response of fifth-order modes by tuning the PID controller in a feedback control system. So^32^ proposed an improved two-degree-of-freedom control framework using the GA algorithm for optimal rectification of the PID controller. After comprehensive analysis, we can deduce that due to the exemplary performance in maturity and stability, GA and PSO algorithms have garnered extensive implementation in practical problem-solving scenarios.

In view of the above study, this paper proposes a fuzzy controller based on an optimisation algorithm aimed at achieving high accuracy and stability of the servo system. A three-loop control simulation model of PMSM in synchronous rotating coordinate system is established from the actual data of ADT-8230 X-axis. The drive is given a position pulse signal command, and the PMSM is driven to the specified target position by the three-loop control model. The position offset command and feedback speed command are obtained by the forward and reverse motion of the motor in the specified range. Due to certain limitations in the traditional PI control method in terms of disturbance rejection and tracking accuracy, we have chosen to adopt fuzzy control as the position loop controller to enhance the system's performance. Within the fuzzy controller, the proportional factor and quantization factor greatly influence the control effectiveness of the system. In order to achieve optimal control results, we have employed an optimization algorithm to fine-tune these two parameters. In practical machining systems, control involves numerous nonlinear issues, and PSO (Particle Swarm Optimization)/GA (Genetic Algorithm) algorithms are well-suited to handle high-dimensional and nonlinear optimization problems. Hence, we have selected the PSO/GA algorithm to optimize the fuzzy controller. Through the use of this optimization algorithm to adjust the proportional factor and quantization factor, we have effectively enhanced the control precision and stability of the system. The optimized fuzzy controller exhibits significant advantages, particularly in terms of high precision and stability. It demonstrates improved robustness and adaptability to environmental changes and external disturbances. By fine-tuning the proportional factor and quantization factor through the optimization algorithm, we have achieved a notable improvement in the system's control precision, enabling the servo system to accurately track specified positions and achieve higher motion accuracy.

## Mathematical modeling of X-axis servo system

### Extracting basic data for ADT-8230

In the process of processing semiconductor chips, dicing error in the micron range is necessary. X-axis is a long-stroke axis, and it carries different speeds for different materials. It brings vibration and response speed, and the size of the speed control range directly affects the dicing quality and processing efficiency. Therefore, in order to ensure the high-speed stability of the linear axis of the machine tool, the servo system is required to have a fast response, a large speed range and a small position tracking error.

The X-axis servo drive and motor are selected from Yaskawa Servo, and the mathematical modelling is mainly based on PMSM. The X-axis servo system adopts three-loop control, and the motion controller is not involved in motion-related instructions, and the three-loop controllers are all in the Yaskawa servo drive. The current loop parameters are not adjustable, and both the velocity and position loops use traditional PI controllers. When the motor is selected, the user does not know the motor's built-in armature resistance and armature inductance. Therefore, RLC bridge measurements are required to derive this value. After calculating the inertia ratio, speed and torque, the X-axis motor power is 400 W, the encoder is a 24-bit linear encoder and the motor is a three-phase AC PMSM.

### Dynamic behavior of PMSM

The three-phase permanent magnet synchronous motor is a strongly coupled, complex nonlinear system, and it is particularly important to establish a suitable mathematical model in order to be able to better design advanced PMSM control algorithms. In this paper, a three-phase PMSM mathematical model is established in a synchronous rotating coordinate system.

The mathematical model of PMSM can be written in the following form: the stator voltage equation is

1 $$ U_d = Ri_d + L_{d}\frac{d}{dt}i_{d} - \omega _{e}L_{q}i_{q} $$

2 $$ U_{q} = Ri_{q} + L_{q}\frac{d}{dt}i_{q} - \omega _{e}(L_{d}i_{d} + \varphi _{f}) $$

where, $U_{d},\;U_{q}$ are the d-q components of the stator voltage; $I_{d},\;I_{q}$ are the d–q axis components of the stator current; $R$ is the resistance of the stator; $\omega e$ is the electric angular velocity; $L_{d},\;L_{q}$ are the d–q axis components of the inductance; $\varphi _{f}$ represents the permanent magnet magnetic chain.

The stator electromagnetic torque equation is

3 $$ T_{e} = 1.5p_{n}i_{q}(i_{d}(L_{d} - L_{q}) + \varphi _{f}) $$

where, $p_{n}$ stands for polarity logarithm.

Transforming the synchronous rotating coordinate system d-q to the natural coordinate system ABC, the variables have the following relationships:

4 $$ \left[ {\begin{array}{*{20}c} {f_A} & {f_B} & {f_C} \\ \end{array} } \right]^{T} = Tc\left[ {\begin{array}{*{20}c} {f_{d}} & {f_{q}} & {f_{0}} \\ \end{array} } \right]^{T} $$

5 $$ Tc = \left[ {\begin{array}{*{20}c} {\cos \theta _{e}} & { - \sin \theta _{e}} & {1/2} \\ {\cos (\theta _{e} - 2\pi /3)} & { - \sin (\theta _{e} - 2\pi /3)} & {1/2} \\ {\cos (\theta _{e} + 2\pi /3)} & { - \sin (\theta_{ e} + 2\pi /3)} & {1/2} \\ \end{array} } \right] $$

where, $Tc$ is the coordinate transformation matrix.

### PMSM conventional three-loop control model

**Figure 2:**
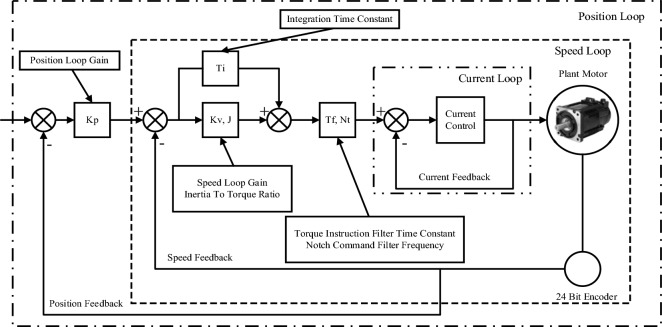
The PMSM three-loop control block diagram.

The X-axis in this study uses the Yaskawa Σ-7 servo drive system, which has an auto-tuning function. Tuning is a function that optimises the responsiveness of the servo unit, and the responsiveness depends on the servo gain set in the servo unit. In general, for machines with high rigidity, responsiveness can be improved by increasing the servo gain. However, for machines with low rigidity, increasing the servo gain may cause vibration and therefore will not improve responsiveness. Servo gain is set by a combination of parameters that interact with each other. The block diagram of the PMSM three loop control is shown in Fig. 2.

The working principle of conventional three-loop control is explained as follows.

First, the upper computer gives the command pulse signal, and the difference between it and the feedback signal is the position error signal. The position loop uses a PI controller, which processes the given error and sends it to the speed loop PI controller. Finally, the signal is transmitted to the current controller for processing to drive the motor rotation. The motor feeds the position signal through a 24-bit linear encoder.

### The scheme of optimization

In this paper, PSO or GA is combined with fuzzy control, mainly considering the following aspects: First, PSO and GA as optimisation algorithms can help the fuzzy control system to perform parameter optimisation and find the best parameter combinations to improve the control effect. Second, PSO and GA can adapt to the characteristics of complex and nonlinear systems to find better control strategies and improve system performance through the global search capability^33^. Third, PSO and GA are adaptive and robust and can adjust the search strategy to maintain system stability in the face of system parameter changes and external disturbances^34^. Fourth, PSO and GA are suitable for multi-objective optimisation problems, and the optimal solution of the system can be obtained through the appropriate objective function trade-off. Therefore, combining PSO or GA with fuzzy control can fully exploit the capability of optimisation algorithms, improve the performance of fuzzy control systems, and adapt to the needs of complex and nonlinear systems^35^.

### Presentation of fuzzy controller design technique

**Figure 3:**
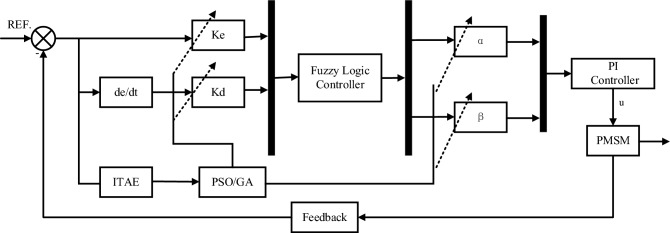
The hybrid structure of the fuzzy position controller.

**Figure 4:**
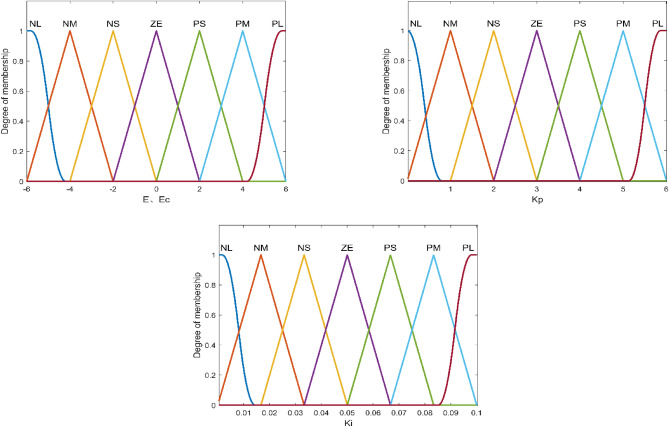
Setup of $e$, $e_{c}$, $K_{p}$ and $K_{i}$ Membership functions and domains.

**Figure 5:**
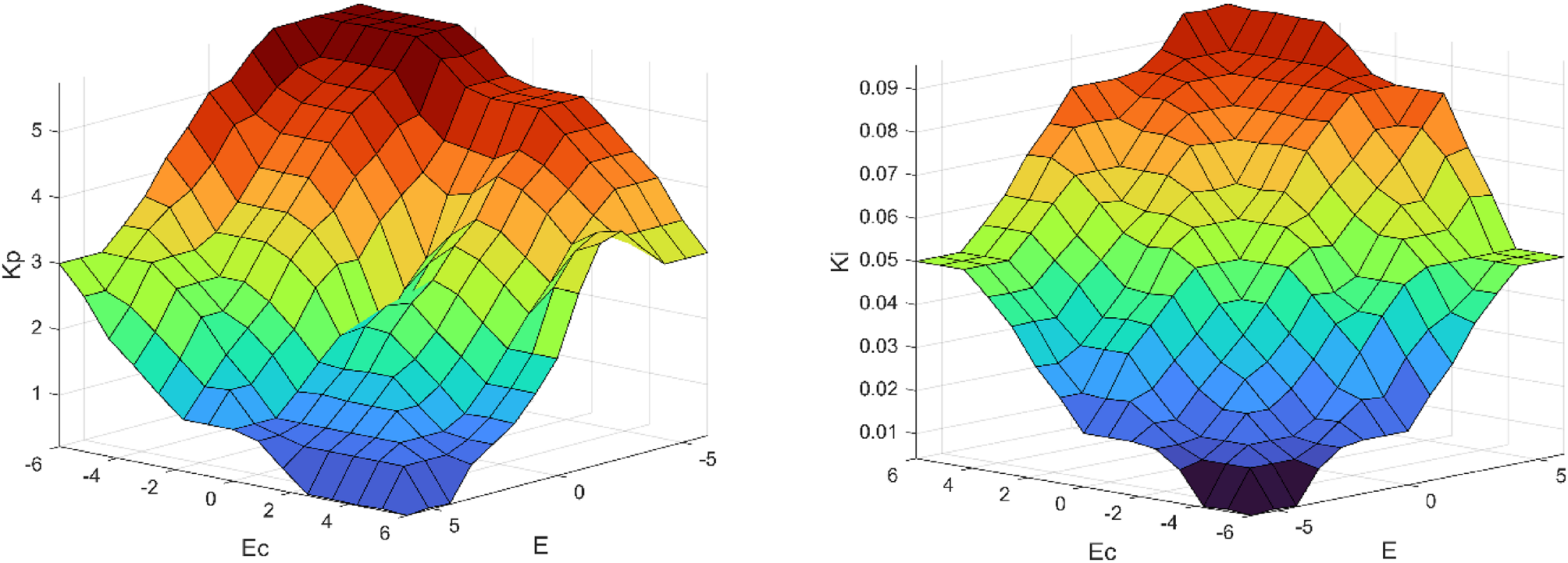
Surface Viewer of rules relationship $e$, $e_{c}$ and $K_{p}$, $K_{i}$.

Fuzzy control technology broadly consists of three parts: fuzzification, fuzzy rules (knowledge base and fuzzy inference), and defuzzification^36^. The hybrid structure of the fuzzy position controller is shown in Fig. 3. In Fig. 3, $K_{e}$, $K_{d}$, $\alpha$ and $\beta$ denoting the scaling factors associated to the inputs and outputs of this hybrid structure. The Membership function editor defines the shape of all the membership functions associated with each variable (Fig. 4). Surface Viewer is used to seeing the dependency of one of the outputs on any one or two inputs. It generates and draws the output surface mapping of the system. (Fig. 5). The fuzzy rule list is given in Tables 1 and 2.

**Table 1:** Fuzzy rule list for $K_{p}$ and $e$, $e_{c}$.

$K_{p}$		$e_c$						
		NL	NM	NS	ZE	PS	PM	PL
$e$	NL	PL	PL	PL	PM	PM	PS	ZE
	NM	PL	PL	PL	PM	PM	PS	ZE
	NS	PL	PM	PM	ZE	PS	ZE	PS
	ZE	PM	PS	PS	NS	ZE	NS	NS
	PS	PS	ZE	ZE	NS	NS	NM	NM
	PM	ZE	ZE	NS	NM	NM	NM	NL
	PL	ZE	NS	NM	NM	NL	NL	NL

**Table 2:** Fuzzy rule list for $K_{i}$ and $e$, $e_{c}$.

$K_{i}$		$e_{c}$						
		NL	NM	NS	NM	PS	PM	PL
$e$	NL	NL	NL	NM	NM	NS	ZE	ZE
	NM	NL	NM	NM	NS	NS	ZE	ZE
	NS	NM	NM	NS	ZE	ZE	PS	PS
	ZE	NM	NS	ZE	ZE	PS	PS	PM
	PS	NS	ZE	ZE	PS	PS	PM	PM
	PM	ZE	ZE	PS	PM	PM	PM	PL
	PL	ZE	ZE	PM	PM	PL	PL	PL

The fuzzy control has two inputs and two outputs; the two inputs are the error and the rate of change of the error. The outputs are $K_{p}$ and $K_{i}$. The inputs and outputs are represented as follows.

6 $$ e(t) = {\text{P}} _{ref} - P_{f}(t) $$

7 $$ e_{c}(t) = \frac{de(t)}{{dt}} $$

8 $$ u = \alpha K_{p} + \beta K_{i} $$

The fuzzy subset and its theoretical domains need to be determined based on the rectification of the conventional PI parameters. The empirical adjustment method is usually used for the traditional PI parameter tuning: First, the adjustment is made in pure proportional action until the system is completely stable. Second, gradually adjust the integration time until stabilization.

The two outputs are $K_{p}$ and $K_{i}$ parameters, respectively. Error $e$ and error rate of change $e_{c}$ can be taken as 7 linguistic variables (NL, NM, NS, ZE, PS, PM, PL) corresponding to negative large, negative medium, negative small, zero, positive small, positive medium, positive large, and the theoretical domain can be determined as $e \in ( - 6,6)$, $e_{c} \in ( - 6,6)$, according to the conventional PI parameter rectification. The theoretical domain of $K_{p}$, $K_{i}$ can be set as $K_{p} \in (0,6)$, $K_{i} \in (0,0.1)$.

$K_{e}$, $K_{d}$, $\alpha$ and $\beta$ denoting the scaling factors associated to the inputs and outputs of this hybrid structure. Determine the argument domain, the quantisation factor and the scaling factor. Let the fundamental domains of $e$,$e_{c}$ be $\left[ { - x_{e} ,x_{e} } \right]$,$\left[ { - x_{ec} ,x_{ec} } \right]$; the fundamental domains of $\Delta K_{p}$, $\Delta K_{i}$ be $\left[ { - y_{p} ,y_{p} } \right]$,$\left[ { - y_{i} ,y_{i} } \right]$; the fuzzy domains of $e$,$e_{c}$ be $\left[ { - m,m} \right]$, $\left[ { - n,n} \right]$; and the fuzzy domains of $\Delta K_{p}$, $\Delta K_{i}$ be $\left[ { - u_{p} ,u_{p} } \right]$, $\left[ { - u_{i} ,u_{i} } \right]$. Then, the quantisation factor $K_{e}$ of $e$, the quantisation factor $K_{d}$ of $e_{c}$, and the scaling factors $\alpha$ and $\beta$ of the output control quantities can be obtained from the following equation:

9 $$ \left\{ \begin{gathered} K_{e} = \frac{m}{{x_{e} }} \hfill \\ K_{ec} = \frac{n}{{x_{ec} }} \hfill \\ \alpha = \frac{{u_{p} }}{{y_{p} }} \hfill \\ \beta = \frac{{u_{i} }}{{y_{i} }} \hfill \\ \end{gathered} \right. $$

The establishment of fuzzy rules is essential. Fuzzy rules are derived from expert knowledge, experience, etc. It is essentially a rule lookup table in the form of "if … and … then …", and given $e$, $e_{c}$ yields 49 different outputs $K_{p}$, $K_{i}$; the key to fuzzy control is rule base, which establishes the rules of fuzzy control, membership function, and fuzzy inference. The minimum operation (Mamdani), which takes the minimal value of the membership function. The membership function using trimf, the output of $K_{p}$ and $K_{i}$ in NL adopts zmf, PL adopts smf, the Mamdani method is used for fuzzy inference, and the center of gravity method is used for defuzzification.

### GA-based fuzzy-PI controller tunning

Because the quantization factor $K_{e}$, $K_{d}$ size on the dynamic performance of the control system has a great impact. $K_{e}$ selected large, the system overshoot is also larger, the transition process is longer, but it can make the rise time shorter; $K_{d}$ selection of the larger, the system overshoot is smaller, but the response speed of the system will be slower, at the same time, $K_{e}$, $K_{d}$ both also interact with each other. $\alpha$, $\beta$ selection of the small will make the system dynamics of the process will be longer, too large and will lead to increased system oscillation, so the determination of the quantisation factor is a cumbersome process. Determining the quantisation factor is therefore a tedious process. Therefore, an optimisation algorithm is introduced to adjust each parameter of the fuzzy control. The fuzzy controller still adjusts the PID parameters $\Delta K_{p}$, $\Delta K_{i}$; the optimisation algorithm optimises the proportionality and quantisation factors of the fuzzy controller. Finally, through the objective function can calculate the adaptation value F, the adaptation value is to judge the current output of the optimisation algorithm PID control parameters is good or bad only standard, through the continuous adjustment of the output PID control parameters, used to reduce the output value of the objective function, so as to achieve the purpose of the optimisation system. The search process for the optimal solution in GA is implemented by using genetic operators that mimic the evolutionary process of living organisms. That is, selection operator, crossover operator, and variation operator^37^. The proportional method of fitness values proposed by Holland^38^ is one of the first selection methods proposed in genetic algorithms. It is a proportional-based selection. If the fitness of an individual $i$ is $f_{i}$_,_ The population size is $N_{p}$_,_ Then the probability of it being selected is expressed as

10 $$ p_{i} = f_{i}/\sum\limits_{i = 1}^{N_{p}} {f_{i}(i = 1,2,...,N_{p})} $$

In order to select crossover individuals, multiple rounds of selection are required to select enough individuals to reach the population size. Generate a uniform random number $r$ within [0,1] in each round. Use $r$ as a selection pointer to determine the selected individual. If $r \le q_{i}$_,_ then individual $i$ is selected; If $q_{k} - 1 < r \le q_{k}(2 \le k \le N_{p})$_,_ then individual $i(i = k)$ is selected. The calculation formula is shown below

11 $$ q_{i} = \sum\limits_{j = 1}^{i} {p_{j}} (i = 1,2,...,N_{p}) $$

where, $qi(i = k)$ is called the accumulation probability of individual $i$.

In this paper, ITAE and is used as a performance index to evaluate the merits of fuzzy controllers, where ITAE is the time multiplied by the integral of the absolute value of the error, expressed as

12 $$ f(t) = \int\limits_{0}^{t} {t\left| {e(t)} \right|} dt $$

**Figure 6:**
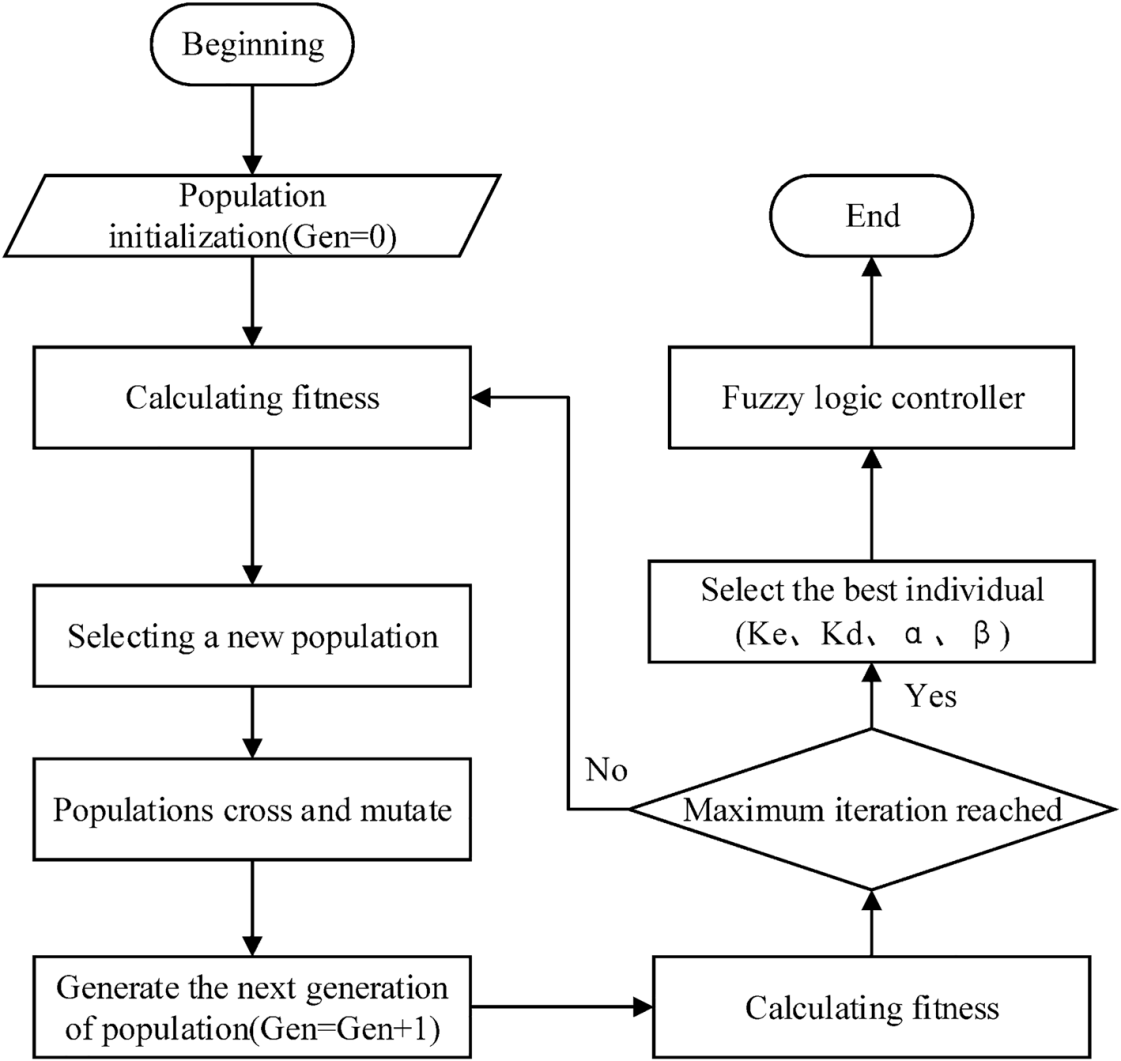
Organigram for GA-fuzzy-PI structure.

The search range for each parameter is;$Ke \in [0,150];Kd \in [0,180];\alpha \in [0,250];\beta \in [0,250]$. Figure 6 shows the flowchart for GA-fuzzy-PI structure.

### PSO-based fuzzy-PI controller tunning

**Figure 7:**
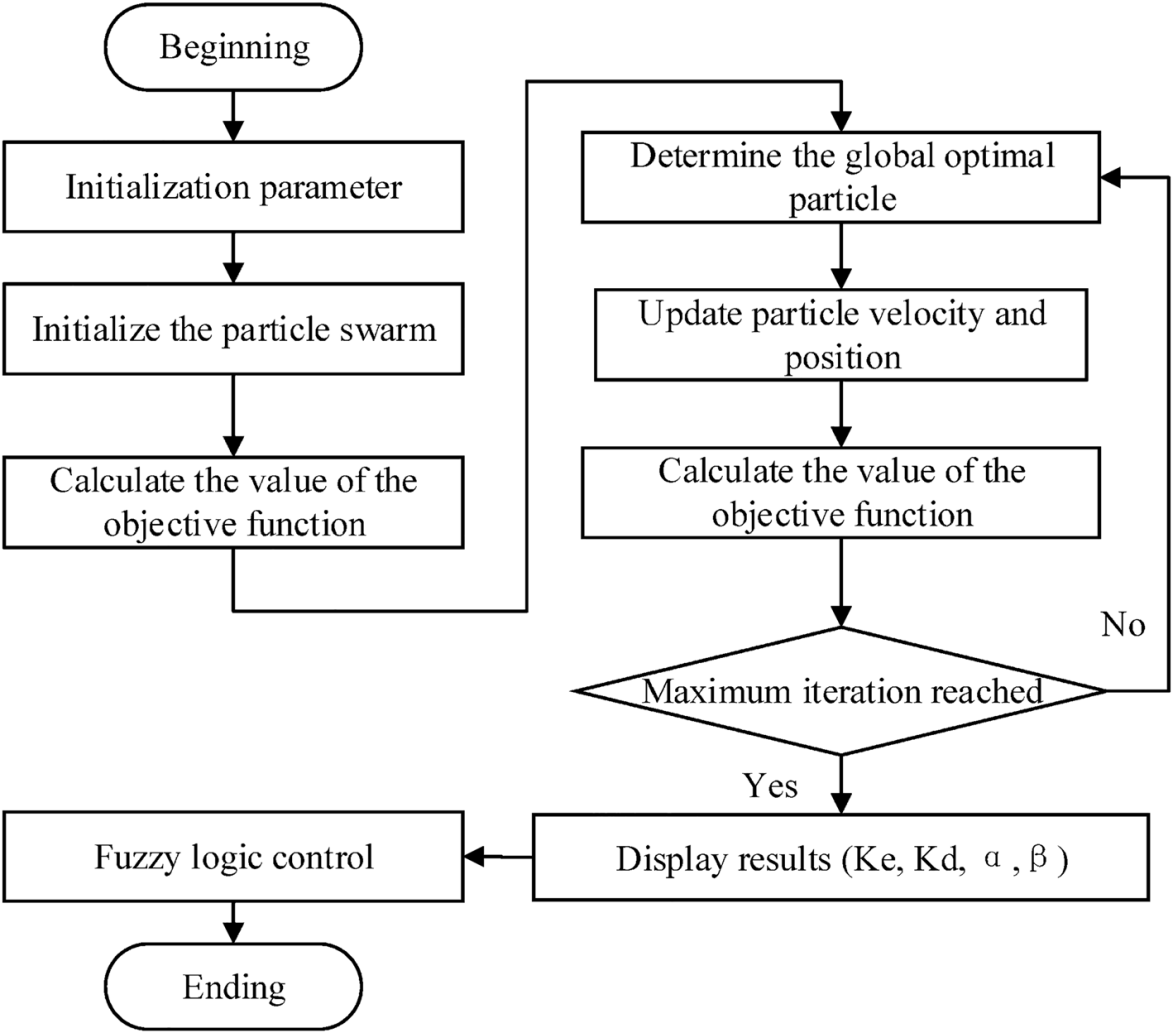
Organigram for PSO-fuzzy-PI structure.

In the process of finding the optimal values, the update rate and position solution of each particle are given by

13 $$ v_{k} + 1 = w \cdot v_{k} + c1 \cdot (pbest_{k} - x_{k}) + c_{2} \cdot (gbest_{k} - x_{k}) $$

14 $$ x_{k} + 1 = x_{k} + v_{k} + 1 $$

where $v_{k}$ is the velocity vector of the particle, $x_{k}$ is the position of the particle, $pbest_{k}$ is the optimal solution position found by the particle itself, and $gbest_{k}$ is the optimal solution position currently found by the whole population. $w$ is the inertia weight. $c_{1}$ and $c_{2}$ are two learning factors, called the "self-knowledge factor" and "social-knowledge factor" of the particle, respectively. These two factors are used to adjust the strength of $pbest_{k}$ and $gbest_{k}$ on particle attraction. $c_{1}$ and $c_{2}$ are in the range of [0,2]. $v_{k}$, $pbest_{k} - x_{k}$ and $gbest_{k} - x_{k}$ are used as the sum of vectors, which is denoted by $v_{k} + 1$. The maximum value of particle velocity in each dimension is less than $v_{max}$. Due to the PSO algorithm has fallen into a local optimal solution. Shi and Eberhart^39^ introduced the inertia weight formula

15 $$ w = w_{\max} - (w_{\max} - w_{\min} )g/G $$

where $g$ generation index represents the current number of evolutionary generations, and $G$ is predefined maximum number of generations. The search range for each parameter is consistent with GA. The flowchart of the PSO-fuzzy-PI structure is shown in Fig. 7.

### Comparison of algorithms

**Figure 8:**
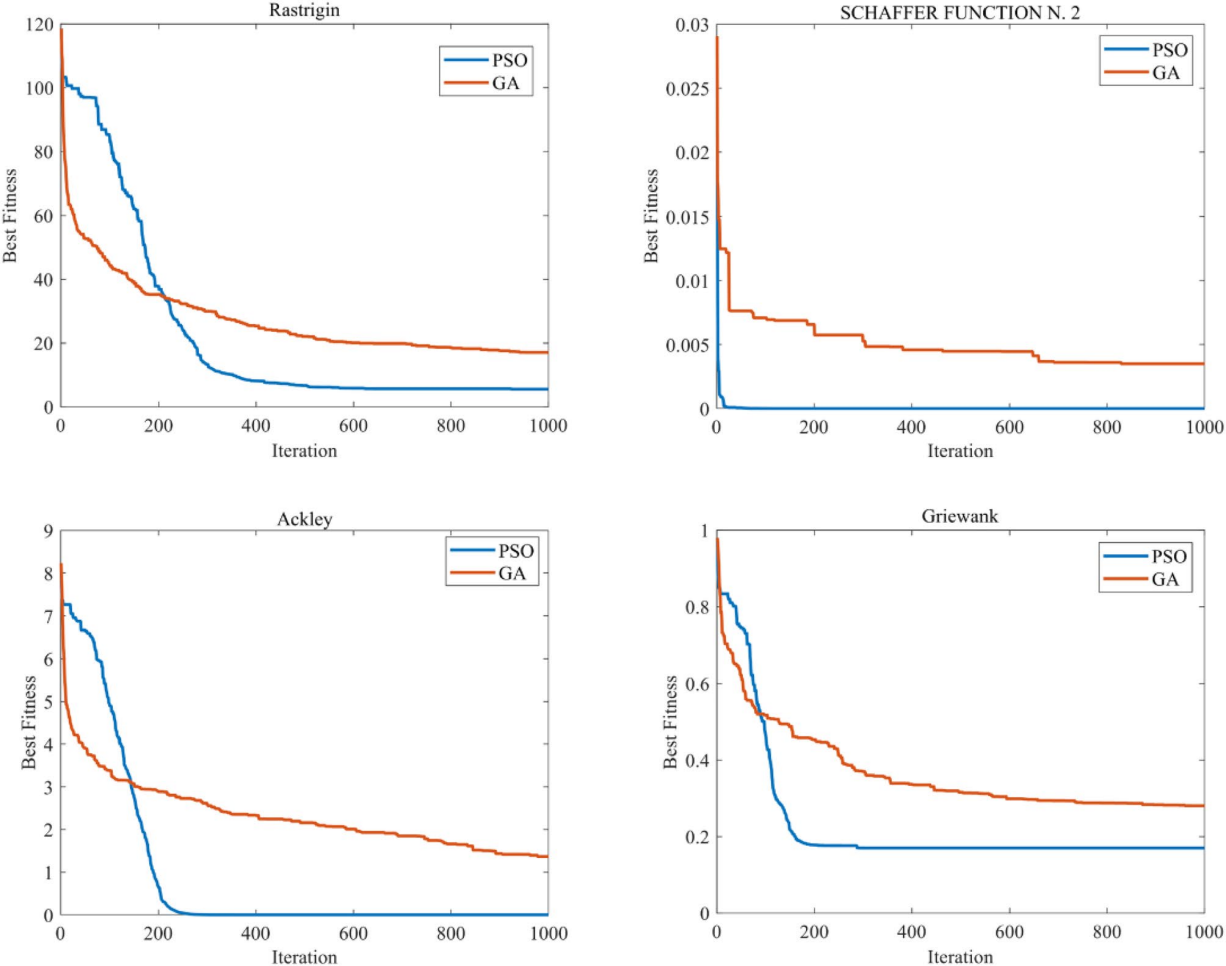
Convergence curve of test function.

**Figure 9:**
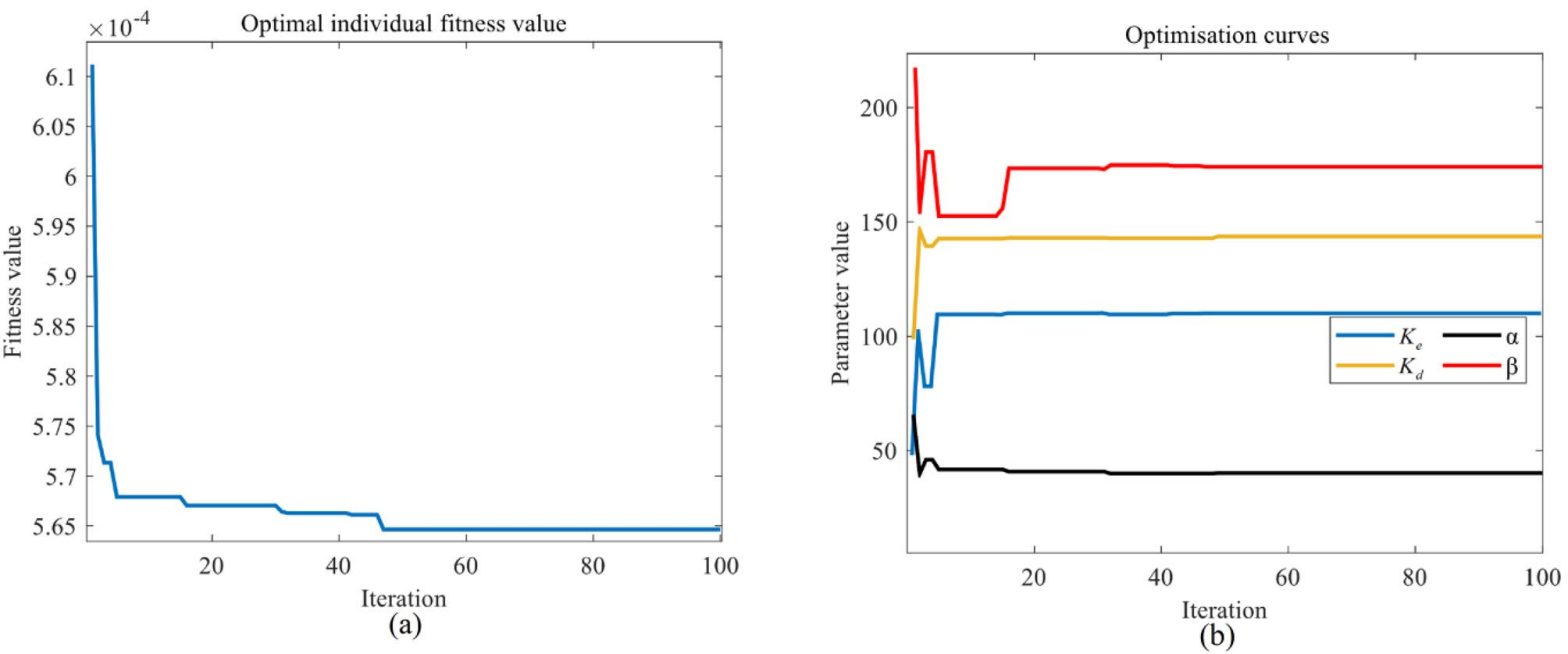
Performance evaluation of the PSO-fuzzy structure: scaling factors and fitness function.

In order to find the optimal algorithm, we selected four sets of test functions to test these two algorithms^40^, the table of test functions is shown in Fig. 9. For both algorithms we set the same number of iterations (1000) and population size (50). In the PSO algorithm the weight factor W was set to 0.6 and the speed factors C1 and C2 were set to 1.414 and 1.632 respectively. In the GA algorithm, the crossover probability, the variation probability and the number of elites were set to 0.6, 0.2 and 5, respectively. The test results are shown in Fig. 8. From the figure we can see that PSO converges faster than GA under either kind of test function, and the time spent.

## Results and discussion

**Figure 10:**
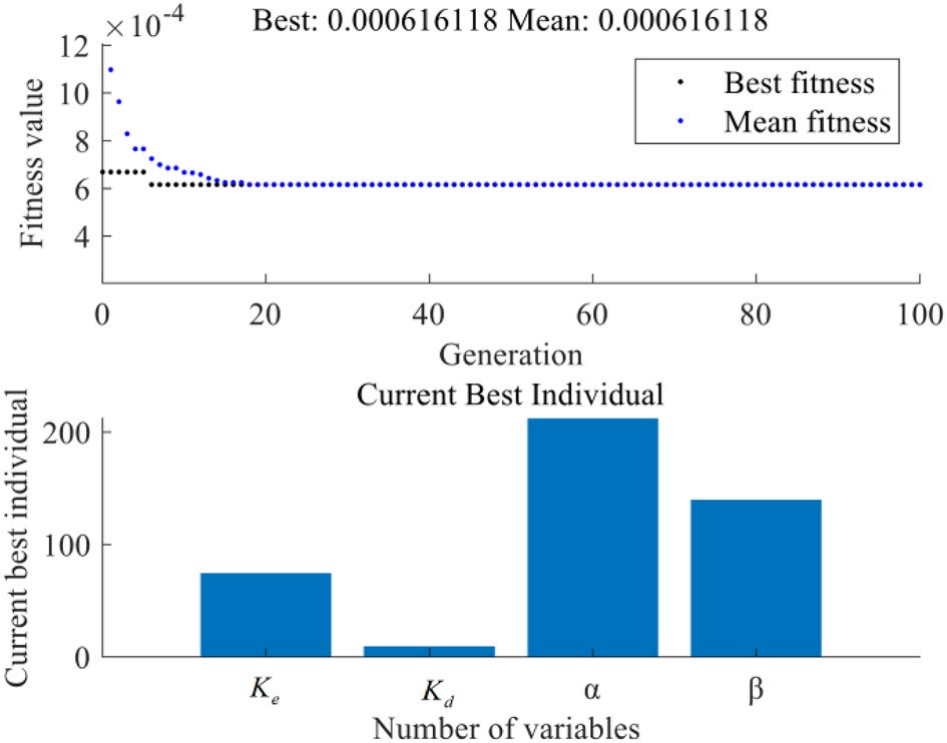
Performance evaluation of the GA-fuzzy structure.

**Figure 11:**
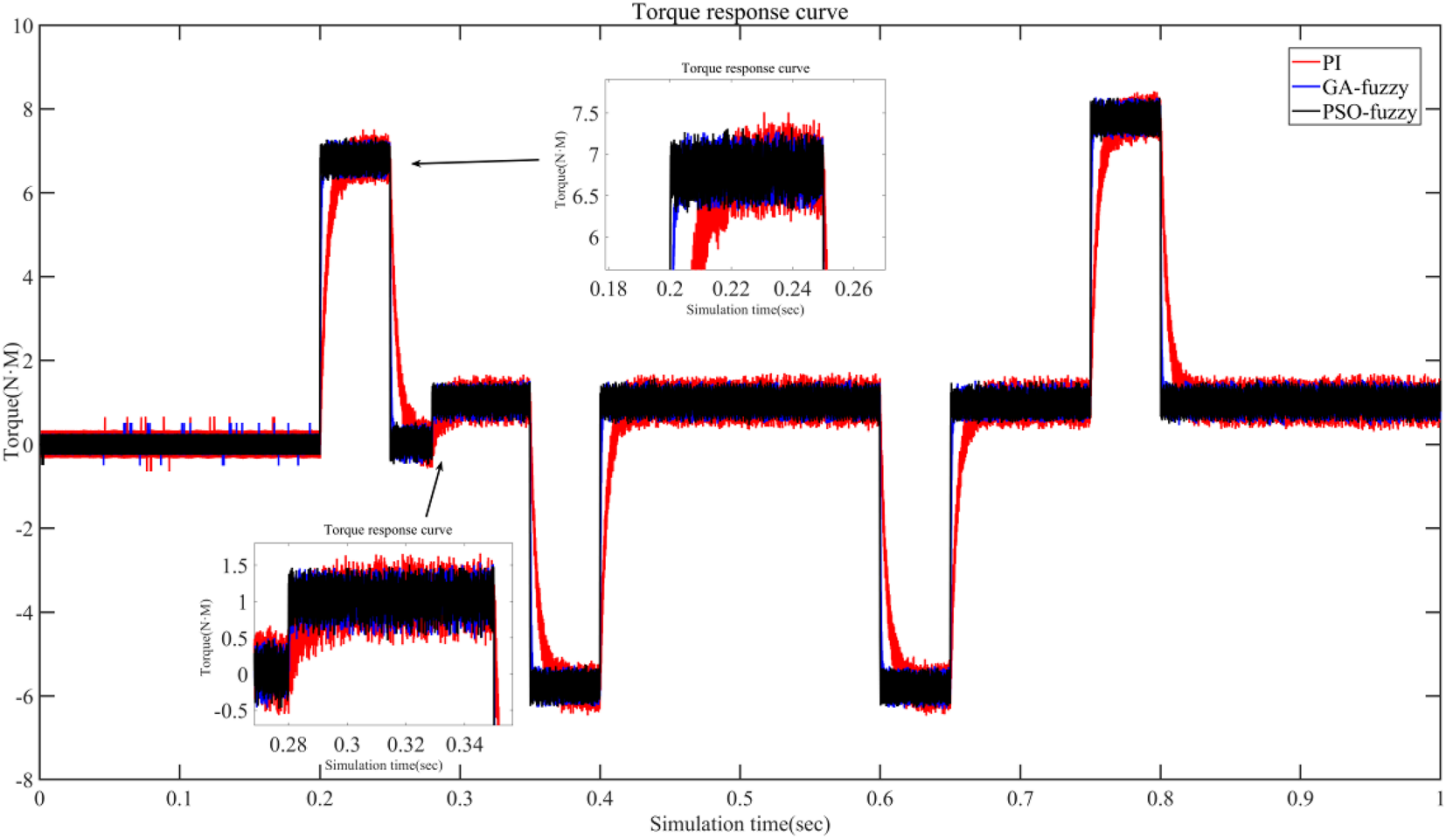
Torque response curve under different conditions.

**Figure 12:**
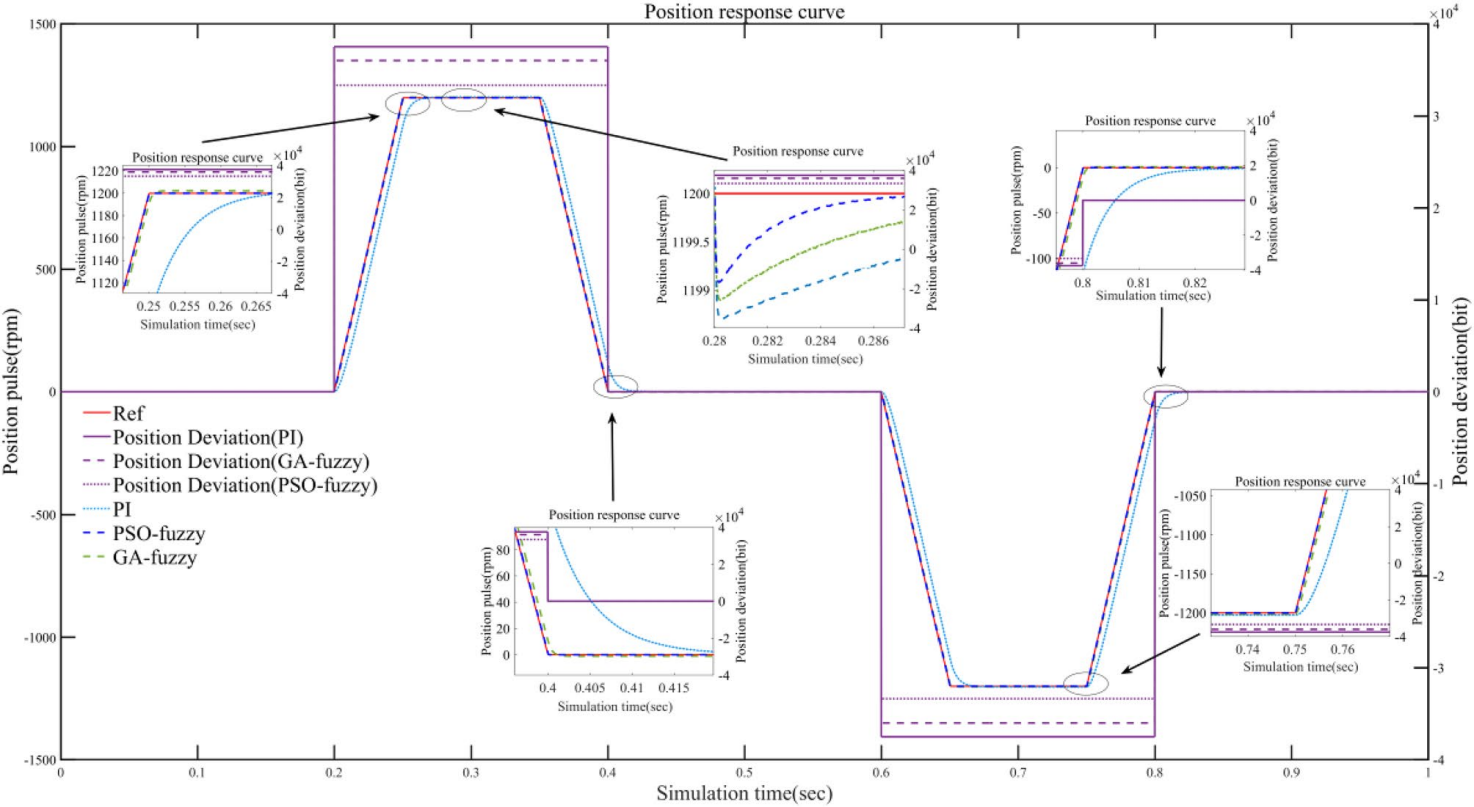
Position-velocity response curve under different conditions.

The Matlab/Simulink platform is used for the implementation of the PSO/GA algorithm combined with the fuzzy controller. The design and analysis of the simulation model relies on M-code and Simulink for offline simulation. To better capture the changes in the system, we set the sampling time to 1e−6. The data of PMSM are given in Table 3. The results of the optimisation search are given in Table 4. The performance evaluation of the PSO-fuzzy/GA-fuzzy structure controllers are shown in Figs. 9 and 10. According to the torque ripple Eq. (16) ^41^, the torque ripple under different control modes is shown in the Fig. 11. The simulation of X-axis servo motion position-velocity curve results in actual engineering is shown in the Fig. 12. Position-velocity response characteristics under different controllers is shown in Table 5.

16 $$ {\text{Torque}}\;{\text{ ripple}}\;{\text{(\% ) = }}\frac{T_{\max} - T_{\min} }{{T_{avg}}} \times 100 $$

**Table 3:** PMSM parameters.

Parameter	Value
Nominal power/KW	0.4
Stator phase resistance (Rs/ohm)	3.965
Stator phase inductance (Ls/H)	10.42e−3
Flux linkage ($\varphi$/Wb)	0.239473
Pole pairs (P)	1
Inertia (J/Kg m^−2^)	4.86e−5
Viscous damping (Kg m^−2^ s^−1^)	3.82e−3
Voltage (V)	400
Rated speed (Rpm)	3000

**Table 4:** Results of the search for excellence.

Controller name	$K_{e}$	$K_{d}$	$\alpha$	$\beta$
GA-fuzzy	74.6458	9.6122	212.248	139.8779
PSO-fuzzy	111.2026	143.6381	40.1157	174.9451

**Table 5:** Position-velocity response characteristics under different controllers.

Controller name	Rise time (s)	Peak_value (rpm)	Peak_overshoot (%)	Torque_ripple (%)	Position error (bit)	Settling time(s)
PI	0.068	1202.7	0.225	36.5	3.48e+4	0.268
GA-fuzzy	0.0549	1201.47	0.1225	30.14	3.18e+4	0.259
PSO-fuzzy	0.0502	1199.98	0	27.98	3.06e+4	0.253

Figures 9 and 10 illustrate the performance evaluation by combining the proposed GA/PSO algorithm with a fuzzy controller. The two methods are determined using the same set of fitness function. Table 4 is the formula for the fitness function, after 100 iterations, the fitness of the fuzzy control optimisssed by PSO is 5.6437e−4 and the fitness of the fuzzy control optimised by GA is 6.161e−4. Table 4 shows the optimisation results of the two control methods. From the illustration, it is apparent that the GA fuzzy control has achieved convergence after 18 iterations, whereas the PSO fuzzy control is capable of escaping local optima even after multiple iterations. This observation signifies that the PSO algorithm possesses superior global search capabilities in solving optimization problems, enabling it to efficiently avoid the constraints of being trapped in local optima. In contrast, the GA algorithm demonstrates convergence in relatively fewer iterations and may be more suitable for simpler optimization problems.

From the Fig. 11 it can be seen that the torque changes at 0.2 s and both have torque pulsation. Under PI control^42^, the torque fluctuation range is 6.15–7.4 N m with a torque pulsation of 1.25 N m; Under GA fuzzy PI control^43^, the torque fluctuation range is 6.308–7.296 N m with a torque pulsation of 0.988N.m; Under PSO fuzzy PI control, the torque fluctuation range is 6.412–7.202 N m with a torque pulsation of 0.792 N m. A disturbance of 3 N m is applied at 0.28 s, so the torque starts to increase from 0.28 s. From the figure it can be seen that the torque response is fastest under PSO fuzzy control, with a torque fluctuation range of 0.732–1.46 N m and a torque pulsation of 0.728 N m; Under GA fuzzy control, torque fluctuation range is 0.592–1.462 N m and torque pulsation is 0.87 N m; Under PI control, the torque fluctuation range is 0.4–1.6 N m and the torque pulsation is 1.2 N m. In summary, PSO fuzzy control has a fast response time and low torque pulsation compared to other control methods.

Figure 12 shows the control action of the three position controllers. The PMSM is set to move back and forth at a given speed of 1200 rpm, with a travel distance of 167,772,160 bit command units and an acceleration and deceleration time of 100 ms. It can be seen that with conventional PI control, the response is slow, reaching the specified speed in 0.26 s and then overshooting. By using GA to tune the fuzzy control parameters, a better dynamic response can be obtained than the conventional PI control, but again with a large overshoot. By using the PSO algorithm to adjust the quantisation factor and the proportionality factor, a good control effect can be obtained with no speed overshoot, good dynamic response and smooth speed. At 0.28 s, an external disturbance of 3N.m is introduced. Based on the observation of the curve graph, the following conclusions can be drawn: traditional PI control method exhibits significant fluctuations during the control process, leading to a substantial decrease in rotational speed, indicating its weak disturbance rejection capability. In contrast, the GA fuzzy control method demonstrates relatively smaller fluctuations. However, the PSO fuzzy control method exhibits the least oscillation, smallest decrease in rotational speed, and shortest stabilization time. The superior performance of the PSO fuzzy control method can be attributed to its combination of fuzzy control and PSO techniques. Fuzzy control enhances the system's disturbance rejection capability, while the PSO algorithm discovers the optimal parameters for the fuzzy controller, further enhancing system stability. Therefore, compared to the traditional PI control method and GA fuzzy control method, the PSO method possesses stronger disturbance suppression ability. It effectively reduces system fluctuations and achieves smaller decreases in rotational speed and shorter stabilization time during the control process. This makes the PSO fuzzy control method an effective control strategy.

According to Table 5, rise time is the time taken for the system to reach the peak state from the initial state. Peak overshoot is the maximum extent to which the system response exceeds the setpoint. Stabilisation time is the time taken for the system to stabilise from the start of the disturbance. Position error is the difference between the system output and the setpoint. PSO-based fuzzy PI controllers have the fastest response, 8% faster than GA fuzzy controllers and 26% faster than conventional PI controllers, and PSO fuzzy controllers have no overshoot, minimise torque ripple and minimise position error. In terms of stabilisation time, there is not much difference between the three controllers, with the PSO fuzzy controller taking the least time and recovering stability the fastest.

## Conclusion

This study presents a strategy for optimizing the X-axis servo system of the dicing saw by replacing the traditional PI control scheme with advanced control strategies. By combining the GA/PSO algorithm with fuzzy logic control, the optimal performance of the position controller is achieved using heuristic optimization techniques. The real data from the ADT-8230 experimental platform is used for modeling, and online simulation experiments are conducted to evaluate the performance of the fuzzy logic and GA/PSO optimized controller. The experimental results demonstrate that the proposed PSO fuzzy structure controller outperforms the traditional PI control method in multiple performance indicators. Compared to PI control, the PSO fuzzy structure controller exhibits better rectification performance, tracking characteristics, and reduced torque ripple. Specifically, the PSO fuzzy structure controller reduces position control error by 11.8%, improves tracking performance by 26%, and reduces torque ripple by 23%. The effectiveness of the proposed method is validated through simulation experiments.

However, as the number of variables in the problem increases, the accuracy and stability of the PSO algorithm may deteriorate. This is because when dealing with more complex optimization problems, the PSO algorithm may quickly converge and get trapped in local optima, failing to find the global optimum. Therefore, in order to enhance the performance of the algorithm, improvements are necessary. In future research, we can integrate the PSO algorithm with other optimization algorithms, leveraging their strengths to further enhance the optimization performance.

Furthermore, due to the confidentiality of the parameters involved in the dicing saw, there are currently some limitations that prevent practical experimentation. In order to overcome these limitations, a potential future research direction is the development of an independent device for the motor controller of the dicing saw, which can be connected to the saw through an Ethernet cable for external control. Through this approach, researchers will be able to manipulate the dicing saw more conveniently and conduct experiments on a broader scale. This will provide researchers with greater flexibility and freedom in their work.

## Data Availability

The datasets used and/or analysed during the current study available from the corresponding author on reasonable request.
